# Serum Orosomucoid Is Associated with Serum Adiponectin, Adipose Tissue Insulin Resistance Index, and a Family History of Type 2 Diabetes in Young Normal Weight Japanese Women

**DOI:** 10.1155/2022/7153238

**Published:** 2022-01-22

**Authors:** Mari Honda, Ayaka Tsuboi, Satomi Minato-Inokawa, Mika Takeuchi, Miki Kurata, Tomofumi Takayoshi, Yushi Hirota, Bin Wu, Tsutomu Kazumi, Keisuke Fukuo

**Affiliations:** ^1^Open Research Center for Studying of Lifestyle-Related Diseases, Mukogawa Women's University, Nishinomiya, Hyogo, Japan; ^2^Department of Health, Sports, and Nutrition, Faculty of Health and Welfare, Kobe Women's University, Kobe, Hyogo, Japan; ^3^Research Institute for Nutrition Sciences, Mukogawa Women's University, Nishinomiya, Hyogo, Japan; ^4^Department of Nutrition, Osaka City Juso Hospital, Osaka, Japan; ^5^Laboratory of Community Health and Nutrition, Department of Bioscience, Graduate School of Agriculture, Ehime University, Matsuyama, Ehime, Japan; ^6^Department of Food Sciences and Nutrition, School of Food Sciences and Nutrition, Mukogawa Women's University, Nishinomiya, Hyogo, Japan; ^7^Division of Diabetes and Endocrinology, Kobe University Graduate School of Medicine, Kobe, Japan; ^8^Department of Endocrinology, First Affiliated Hospital of Kunming Medical University, Kunming, Yunnan, China; ^9^Department of Medicine, Kohnan Kakogawa Hospital, Kakogawa, Hyogo, Japan

## Abstract

**Introduction:**

Adipose tissue (AT) expandability may be facilitated by adiponectin and suppressed by orosomucoid, and reduced AT expandability may be associated with first-degree relatives of type 2 diabetes. We tested the hypothesis that orosomucoid may be associated not only with adiponectin and adipose tissue insulin resistance but also with a family history of type 2 diabetes (FHD). *Research Design and Methods.* Anthropometric and metabolic variables, adipokines, and measures of inflammatory and insulin resistance were cross-sectionally investigated in 153 young normal weight Japanese women. Stepwise multivariate linear regression analyses were used to identify the most important determinants of orosomucoid.

**Results:**

Orosomucoid was higher in women with positive (*n* = 57) compared to women with negative FHD and was associated positively with FHD (both *p* = 0.01). Orosomucoid also showed positive associations with fasting glucose (*p* < 0.001), free fatty acids (*p* = 0.001), and HbA1c (*p* = 0.007), whereas there was no association with fasting insulin and serum lipids. In addition, orosomucoid was associated inversely with adiponectin (*p* = 0.02) and positively with adipose tissue-insulin resistance index (AT-IR, the product of fasting insulin and free fatty acids; *p* = 0.001) but not with homeostasis model assessment-insulin resistance, leptin, and high-sensitivity C-reactive protein. In multivariate analyses, AT-IR (standardized *β*, 0.22; *p* = 0.003), serum adiponectin (standardized *β*, -0.163; *p* = 0.032), FHD+ (standardized *β*, 0.178; *p* = 0.029), and HbA1c (standardized *β*, 0.213; *p* = 0.005) emerged as independent determinants of orosomucoid and explained 15.2% of its variability.

**Conclusions:**

These results are the first to demonstrate that orosomucoid is associated not only with adipose tissue-insulin resistance and adiponectin but also with FHD.

## 1. Introduction

Orosomucoid (ORM), also known as *α*-1 acid glycoprotein, is one of the acute-phase proteins with a variety of biological activities [1]. Although it is mainly synthesized by the liver, adipocytes are capable of producing ORM, suggesting ORM as an adipokine [2]. In subjects under elective abdominal surgery for cholecystectomy and weight reduction surgery whose BMI averaged 40 kg/m^2^ [3], the level of circulating ORM correlated positively with BMI, body fat mass, and serum leptin. It also correlated with fasting insulin, homeostasis model assessment-insulin resistance (HOMA-IR) values, and C-reactive protein (CRP) in men. Further, ORM mRNA expression correlated with mRNA expression of adiponectin in visceral and subcutaneous adipose tissue [3]. Higher circulating levels of ORM and CRP have been reported to be associated with an increased risk of type 2 diabetes and cardiovascular diseases [4]. We have previously shown that elevated circulating ORM was associated with elevated glucose excursion during an oral glucose tolerance test in young and middle-aged Japanese people [5–7].

Family history of type 2 diabetes (FHD) is a well-known risk factor of the disease. Studies on association between FHD and inflammatory markers are limited, and results are inconsistent. Elevated CRP concentrations were associated with FHD in adult women whose BMI averaged > 30 kg/m^2^ [8]. However, CRP concentrations were not associated with FHD in young and middle-aged first-degree relatives of type 2 diabetic subjects [9, 10]. As far as we know, studies are missing on association of FHD with ORM.

Studies have shown that individuals with genetic predisposition for type 2 diabetes (first-degree relatives) were characterized by restricted adipogenesis [11, 12]. It has recently been shown that ORM secretion induced by bile acids from the liver suppresses adipogenesis [13]. In contrast, adiponectin has been shown to stimulate AT expandability [14]. We have recently found that young normal weight Japanese women with FHD had lower subcutaneous (limb and gluteal) fat mass, a subtle lipodystrophy-like phenotype and elevated ORM [15]. In the present study, therefore, we tested the hypothesis that orosomucoid may be associated not only with adipose tissue-insulin resistance and adiponectin but also with FHD.

## 2. Methods

We cross-sectionally studied 153 women who provided FHD data among 168 women whose details were previously reported elsewhere [5, 15]. Fifty-seven women reported that a parent or a grandparent were on antidiabetic drugs; they were considered FHD positive (FHD+), and 96 gave no family history of the disease (FHD-). Unfortunately, information was not available on the extent of family history (i.e., how many family members have the condition) and the nature of the family history (paternal or maternal). There were no significant differences in anthropometric and biochemical measurements between 153 women studied and 15 women whose FHD data were not available (data not shown). Participants were students of the Mukogawa Women's University and recruited as volunteers. Subjects with clinically diagnosed acute or chronic inflammatory diseases, endocrine, cardiovascular, hepatic, renal diseases, hormonal contraception, and unusual dietary habits were excluded. Nobody reported to receive any medications or have regular supplements. The study was approved by the Ethics Committees of the Mukogawa Women's University (No. 07-28 on 19/02/2008) to be in accordance with the Helsinki declaration. All subjects gave written consent after the experimental procedure had been explained.

After a 12 h overnight fast at 8:30 A.M., the height, weight, and waist were measured. Blood was drawn from the cubital vein and HbA1c, glucose, insulin, serum and high-density lipoprotein (HDL) cholesterol, triglycerides, free fatty acid (FFA), adiponectin, leptin, ORM, and high-sensitivity CRP (hsCRP) were measured as previously reported [16]. Homeostasis model assessment-insulin resistance (HOMA-IR) was calculated as the product of fasting insulin and glucose [17]. Insulin resistance in adipose tissue was evaluated by adipose tissue-insulin resistance (AT-IR), which is calculated by the product of fasting insulin and FFA [18]. We have recently shown that AT-IR may be a simple and useful surrogate index of adipose tissue insulin resistance even in Japanese women without diabetes and obesity [19].

Fat mass, bone mass, and lean mass for arms, legs, trunk, and the total body were measured using whole-body dual-energy X-ray absorptiometry (DXA) (Hologic QDR-2000, software version 7.20D, Waltham, MA) as previously reported [16]. The leg region included the entire hip, thigh, and leg. General adiposity was assessed by BMI and fat mass index (FMI) calculated as body fat mass in kg divided by height in meter squared. Abdominal fat accumulation was assessed by waist and the ratio of trunk fat to leg fat [20].

Data were presented as mean ± SD. Due to deviation from normal distribution, HOMA-IR and hsCRP were logarithmically transformed for analyses. Differences between FHD+ and FHD- were compared by *t* test. Correlations of ORM with metabolic parameters were evaluated by Pearson's correlation analyses. Stepwise multiple linear regression analyses were performed to further identify the most significant variables contributing to the variation of ORM. Independent variables included were those that displayed significant associations with ORM. HbA1c, instead of fasting glucose, was included because studies including ours have shown a strong association with fasting glucose [5, 21–23]. A two-tailed value of *p* < 0.05 was considered significant. Statistics were performed with SPSS system 17.0 (SPSS Inc., Chicago, IL).

## 3. Results

Waist averaged < 75 cm, fasting glucose < 85 mg/dL, triglyceride < 60 mg/dL, insulin < 6 *μ*U/mL, and HDL cholesterol > 70 mg/dL and did not differ between 57 FHD+ and 96 FHD- (Table 1). FHD+ compared with FHD- had elevated ORM. Post hoc power analyses for ORM comparison yielded 0.727, indicating a slightly weak but satisfactory statistical power, in the setting of the present study although no statistical sample size calculations were conducted. Trunk/leg fat ratio, percentage body fat, and FMI did not differ although BMI was tended to be lower in FHD+. FFA and systolic and diastolic BP were tended to be higher in FHD+. Other variables studied in the present study did not differ between the two groups of women. As we believe that women who did not provide data on FHD might not have FHD, we did the same analyses described above, in which fifteen women who did not provide data on FHD were included as negative FHD (online supplementary table 1). Results were essentially the same as in Table 1.

**Figure 1:**
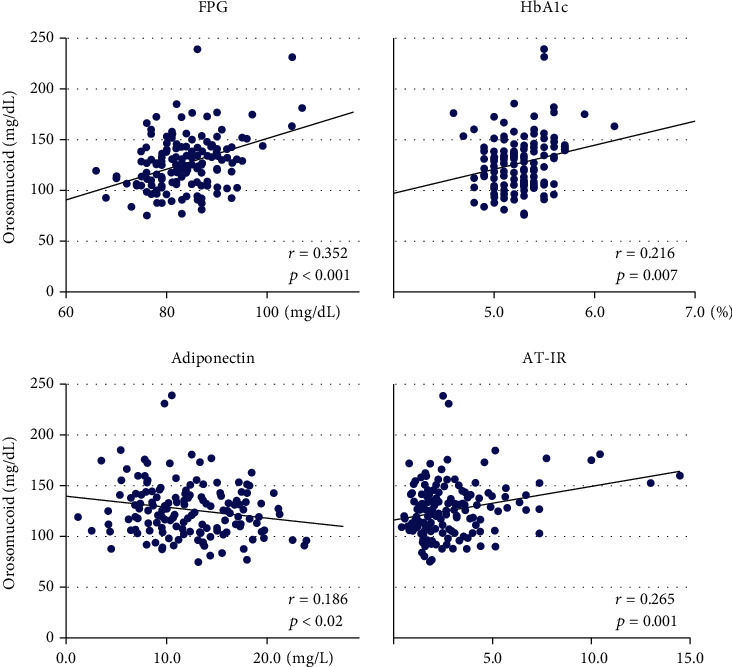
The scatter plot between serum orosomucoid and fasting plasma glucose (FPG), HbA1c, serum adiponectin, and adipose tissue-insulin resistance index (AT-IR).

ORM showed positive associations with fasting glucose (Figure 1) and FFA but not with fasting insulin (Table 1). However, the association with AT-IR (Figure 1) was significant whereas the association with HOMA-IR was not. ORM was also associated positively with HbA1c and inversely with adiponectin (Figure 1). ORM was associated with FHD (*r* = 0.208, *p* = 0.01) although there was no association with anthropometric indices, leptin, hsCRP, and blood pressure.

We then have done multivariate linear regression analyses for ORM as a dependent variable and FHD+, HbA1c, AT-IR, and adiponectin as independent variables (Table 2). In addition to HbA1c, AT-IR, adiponectin, and FHD+ emerged as independent determinants of ORM. These four variables explained 15.2% of the ORM variability. We previously showed strong associations between ORM and 30 min glucose (*r* = 0.919, *p* < 0.001) and the area under the glucose concentration curve (*r* = 0.915, *p* < 0.001) during oral glucose tolerance tests [5]. Therefore, we have done multivariate linear regression analyses which included either of the two glycemic variables as an additional independent variable. Either of the two but none of the four variables in the original model emerged as an independent determinant for ORM (data not shown).

As we have recently reported an independent association between FHD and birth weight [21], we made multivariate logistic regression analyses for FHD including birth weight and ORM as independent variables. FHD was marginally associated with birth weight (odds ratio: 0.999, 95% confidential interval: 0.998-1.000, *p* = 0.051) but not with ORM (odds ratio: 1.012, 95% confidential interval: 0.995-1.029, *p* = 0.16).

## 4. Discussion

Our study demonstrates that serum adiponectin was an independent contributor to ORM in nonobese young normal weight Japanese women. AT-IR but not HOMA-IR was an independent contributor to ORM as well. Further, FHD was associated with ORM. The association with HbA1c in the present study may be consistent with previously reported associations with glycemia [5, 7, 22–24]. It is worth noting that young women were normal weight, rather lean, normoglycemic, normolipidemic, and were not insulin resistant. In addition, we studied Japanese female students of Mukogawa Women's University, where more than 90% of grade 1 students are 18 years old [16]. This approach may decrease the interference of age and environmental factors, including smoking, alcohol, educational, and socioeconomic status. [16].

ORM, one of the most abundant proteins, has a variety of activities [1]. It is well-known that the glycoprotein is an acute-phase reactant and disease marker. ORM also has the ability to bind and to carry drugs. ORM can also maintain the barrier function of capillary and mediate the sphingolipid metabolism [1]. It has recently been shown that ORM suppresses adipogenesis [13]. Genome-wide association studies have shown that restricted adipogenesis was characteristic of individuals with first-degree relatives with type 2 diabetes [11, 12]. These findings may be consistent with our finding that ORM was independently associated with FHD in young Japanese women.

Studies on association between ORM and markers of insulin resistance are limited, and again, results are inconsistent. In subjects with morbid obesity [3], ORM correlated positively with fasting insulin and HOMA-IR in men but not in women. It was also correlated with BMI, body fat mass, and serum leptin, suggesting that adipose tissue may be a contributing source for circulating ORM [2]. In the Atherosclerosis Risk in Communities Study, [22] ORM showed positive associations with both fasting glucose and insulin although results with HOMA-IR were not reported. ORM has recently been shown to suppress adipogenesis [13] and reduced adipogenesis may lead to hypertrophic, dysfunctional, and insulin-resistant adipocytes [25]. These observations may be related to the independent association of ORM with adipose tissue insulin resistance index but not with HOMA-IR, a surrogate marker of hepatic insulin resistance [16].

After weight reduction in obese subjects, elevated serum ORM was decreased [26–28] and decreased serum adiponectin was increased [29]. However, in subjects with a mean BMI of 40 kg/m^2^ [3], there was no association between circulating ORM and adiponectin although there was a positive correlation between ORM and adiponectin mRNA expression in both visceral and subcutaneous adipose tissues. Adiponectin can facilitate AT expansion [14] whereas ORM may suppress adipogenesis [13]. Studies have shown that serum adiponectin was lower in children and adults with FHD and was associated with FHD [30–33]. Therefore, we speculate that the inverse and independent association between ORM and adiponectin in the present study may suggest a regulatory role for ORM and adiponectin in glucose and lipid homeostasis through the regulation of adipogenesis and/or AT expandability.

Studies on associations between FHD and inflammatory markers are also limited, and results are inconsistent. Elevated CRP concentrations were associated with FHD in adult women whose BMI averaged > 30 kg/m^2^ [9]. However, CRP concentrations were not associated with FHD in young and middle-aged first-degree relatives of type 2 diabetic subjects [10, 11, 33–35]. To the best of our knowledge, the present study is the first to demonstrate the association between FHD and ORM.

There are a lot of limitations in the present study. They include the cross-sectional design of the present study and a single measurement of biochemical variables. The recruitment procedure may also have impact on the results. As the participation was voluntary, women who pay more attention to health may be more likely to participate. We used crude measures of insulin sensitivity/insulin resistance, which may be less accurate. Statistical power was not calculated. As we studied young Japanese women only, results may not be generalized to other gender, age populations, races, or ethnicities. A homogeneous study population with scarce confounding factors as previously reported [15] and accurate and reliable measures of DXA-derived body composition are the strengths of the present study.

In conclusion, the present study has demonstrated that ORM was independently associated not only with serum adiponectin (inversely) and adipose tissue insulin resistance but also with FHD, in addition to HbA1c. As ORM, adiponectin and FHD all seem to be associated with adipogenesis and/or AT expandability, reduced adipogenesis, and/or reduced AT expandability associated with adipose tissue insulin resistance, may be a knot of these associations in young normal weight Japanese women. Further studies should be performed in other populations to confirm the association between ORM and FHD.

## Figures and Tables

**Table 1:** Orosomucoid serum concentrations and other characteristics of 153 young Japanese women with and without a family history of type 2 diabetes and correlation coefficient (*r*) of orosomucoid.

	Family history of diabetes		*p* values	*r*	*p* values
	Yes (*n* = 57)	No (*n* = 96)			
Orosomucoid (mg/dL)	134 ± 30	122 ± 24	0.010	1	—
Age (years)	20.5 ± 1.0	20.4 ± 1.1	0.536	0.145	0.073
Body mass index (kg/m^2^)	20.3 ± 2.9	21.0 ± 2.1	0.064	-0.039	0.629
Waist (cm)	72.6 ± 5.5	73.6 ± 6.0	0.323	0.011	0.889
Trunk/leg fat ratio	1.24 ± 0.25	1.24 ± 0.24	0.909	0.046	0.569
Percentage body fat (%)	25.7 ± 6.8	26.2 ± 5.7	0.640	-0.032	0.693
Fat mass index (kg/m^2^)	5.27 ± 2.03	5.50 ± 1.57	0.431	-0.044	0.593
Fasting glucose (mg/dL)	84 ± 8	83 ± 6	0.572	0.352	<0.001
Fasting insulin (*μ*U/mL)	5.6 ± 3.2	5.9 ± 3.2	0.653	0.068	0.403
Free fatty acids (mEq/L)	0.56 ± 0.25	0.49 ± 0.21	0.064	0.263	0.001
HbA1c (%)	5.2 ± 0.2	5.2 ± 0.2	0.921	0.216	0.007
HOMA-IR	1.2 ± 0.8	1.2 ± 0.7	0.833	0.130	0.110
AT-IR	3.2 ± 2.7	2.8 ± 1.8	0.259	0.265	0.001
Triglycerides (mg/dL)	60 ± 56	57 ± 21	0.577	0.025	0.762
Total cholesterol (mg/dL)	181 ± 30	187 ± 27	0.249	-0.002	0.981
HDL cholesterol (mg/dL)	71 ± 11	75 ± 13	0.115	-0.033	0.689
Leptin (ng/mL)	7.0 ± 3.6	8.0 ± 3.8	0.130	0.058	0.478
Adiponectin (mg/L)	12.0 ± 5.0	12.5 ± 4.5	0.509	-0.186	0.021
hsCRP (*μ*g/dL)	36 ± 76	32 ± 62	0.699	-0.033	0.687
Systolic BP (mmHg)	112 ± 13	109 ± 12	0.081	0.062	0.446
Diastolic BP (mmHg)	67 ± 8	65 ± 7	0.077	0.024	0.771

Mean ± SD. HDL: high-density lipoprotein; HOMA-IR: homeostatic model assessment-insulin resistance; AT-IR: adipose tissue-insulin resistance; hsCRP: high-sensitivity C-reactive protein; BP: blood pressure.

**Table 2:** Stepwise multiple regression analysis for serum orosomucoid as a dependent variable.

	Standardized *β*	Cumulative *R*^2^	*p* values
FHD+	0.178	0.029	0.019
HbA1c	0.213	0.067	0.005
AT-IR	0.228	0.131	0.003
Adiponectin	-0.163	0.152	0.032

Independent variables included were positive family history of diabetes (FHD+) and variables that showed significant associations with orosomucoid in Table 1: HbA1c, adipose tissue insulin resistance (AT-IR), and adiponectin.

## Data Availability

The ethics committee of the University does not allow us to open data except for a manuscript.

## Abbreviations

AT-IR: Adipose tissue-insulin resistance
CRP: C-reactive protein
DXA: Dual-energy X-ray absorptiometry
FFA: Free fatty acid
FHD: Family history of type 2 diabetes
FMI: Fat mass index
HDL: High-density lipoprotein
hsCRP: High-sensitivity CRP
HOMA-IR: Homeostasis model assessment-insulin resistance
ORM: Orosomucoid.
