# Supplementary material for: Serum Orosomucoid Is Associated with Serum Adiponectin, Adipose Tissue Insulin Resistance Index, and a Family History of Type 2 Diabetes in Young Normal Weight Japanese Women
Source: J Diabetes Res. 2022 Jan 22;2022:7153238. doi: 10.1155/2022/7153238 (PMC8800618; doi:10.1155/2022/7153238)
Supplement: Supplementary Materials [file 7153238.f1.docx]

| Supplementary table 1. Orosomucoid serum concentrations and other characteristics of 168 young Japanese women with and without a family history of type 2 diabetes | | | | | | | |
| --- | --- | --- | --- | --- | --- | --- | --- |
|  | Family history of diabetes | | | | | |  |
|  | Yes (n=57) | | | No (n=111) | | | p values |
| Orosomucoid (mg/dL) | 134 | ± | 30 | 123 | ± | 24 | .014 |
| Age (years) | 20.5 | ± | 1.0 | 20.4 | ± | 1.1 | .436 |
| Body mass index (kg/m2) | 20.3 | ± | 2.9 | 21.0 | ± | 2.1 | .046 |
| Waist (cm) | 72.6 | ± | 5.5 | 73.5 | ± | 5.7 | .303 |
| Trunk/leg fat ratio | 1.24 | ± | 0.25 | 1.22 | ± | 0.24 | .629 |
| Percentage body fat (%) | 25.7 | ± | 6.8 | 25.8 | ± | 5.9 | .942 |
| Fat mass index (kg/m2) | 5.27 | ± | 2.03 | 5.41 | ± | 1.57 | .613 |
| Fasting glucose (mg/dL) | 84 | ± | 8 | 84 | ± | 6 | .667 |
| Fasting insulin (μU/mL) | 5.6 | ± | 3.2 | 5.9 | ± | 3.1 | .642 |
| Free fatty acids (mEq/L) | 0.56 | ± | 0.25 | 0.49 | ± | 0.21 | .058 |
| HbA1c (%) | 5.2 | ± | 0.2 | 5.2 | ± | 0.2 | .976 |
| HOMA-IR | 1.19 | ± | 0.77 | 1.22 | ± | 0.63 | .815 |
| AT-IR | 3.20 | ± | 2.66 | 2.73 | ± | 1.72 | .230 |
| Triglycerides (mg/dL) | 60 | ± | 56 | 56 | ± | 21 | .523 |
| Total cholesterol (mg/dL) | 181 | ± | 30 | 187 | ± | 29 | .240 |
| HDL cholesterol (mg/dL) | 71 | ± | 11 | 75 | ± | 13 | .052 |
| Leptin (ng/mL) | 7.0 | ± | 3.6 | 7.8 | ± | 3.8 | .210 |
| Adiponectin (mg/L) | 12.0 | ± | 5.0 | 12.5 | ± | 4.4 | .485 |
| hsCRP (μg/dL) | 36 | ± | 76 | 31 | ± | 59 | .648 |
| Systolic BP (mmHg) | 112 | ± | 13 | 108 | ± | 11 | .056 |
| Diastolic BP (mmHg) | 67 | ± | 8 | 65 | ± | 7 | .076 |
| Mean±SD Abbreviations are the same as in Table 1 | | | | | | | |
